# Evidence for Reduced Malaria Parasite Population after Application of Population-Level Antimalarial Drug Strategies in Southern Province, Zambia

**DOI:** 10.4269/ajtmh.19-0666

**Published:** 2020-07-02

**Authors:** Rachel F. Daniels, Stephen F. Schaffner, Adam Bennett, Travis R. Porter, Joshua O. Yukich, Conceptor Mulube, Brenda Mambwe, Mulenga C. Mwenda, Sandra Chishimba, Daniel J. Bridges, Hawela Moonga, Busiku Hamainza, Elizabeth Chizema Kawesha, John M. Miller, Richard W. Steketee, Dyann F. Wirth, Thomas P. Eisele, Daniel L. Hartl, Sarah K. Volkman

**Affiliations:** 1 Harvard T.H. Chan School of Public Health, Boston, Massachusetts;; 2 The Broad Institute of MIT and Harvard, Cambridge, Massachusetts;; 3 Malaria Elimination Initiative, Global Health Group, University of California San Francisco, San Francisco, California;; 4 Center for Applied Malaria Research and Evaluation, Tulane University School of Public Health and Tropical Medicine, New Orleans, Louisiana;; 5 PATH Malaria Control and Elimination Partnership in Africa (MACEPA), Lusaka, Zambia;; 6 National Malaria Elimination Centre, Zambia Ministry of Health, Lusaka, Zambia;; 7 PATH MACEPA, Seattle, Washington;; 8 Harvard University, Cambridge, Massachusetts;; 9 Simmons University, Boston, Massachusetts

## Abstract

A mass drug administration trial was carried out in Southern Province, Zambia, between 2014 and 2016, in conjunction with a standard of care package that included improved surveillance, increased access to malaria case management, and sustained high levels of vector control coverage. This was preceded by mass test and treatment in the same area from 2011 to 2013. Concordant decreases in malaria prevalence in Southern Province and deaths attributed to malaria in Zambia over this time suggest that these strategies successfully reduced the malaria burden. Genetic epidemiological studies were used to assess the consequences of these interventions on parasite population structure. Analysis of parasite material derived from 1,620 rapid diagnostic test (RDT)-positive individuals obtained from studies to evaluate trial outcomes revealed a reduction in the average complexity of infection and consequential increase in the proportion of infections that harbored a single parasite genome (monogenomic infections). Highly related parasites, consistent with inbreeding, were detected after interventions were deployed. Geographical analysis indicated that the highly related infections were both clustered focally and dispersed across the study area. These findings provide genetic evidence for a reduced parasite population, with indications of inbreeding following the application of comprehensive interventions, including drug-based campaigns, that reduced the malaria burden in Southern Province. Genetic data additionally revealed the relationship between individual infections in the context of these population-level patterns, which has the potential to provide useful data for stratification and targeting of interventions to reduce the malaria burden.

## INTRODUCTION

Population-level drug-based interventions including mass drug administration (MDA) and mass test and treatment (MTAT) have been evaluated with a goal to reduce the malaria burden in a number of settings.^1,2^ Current recommendations of the WHO^3^ include the use of MDA to interrupt transmission of *Plasmodium falciparum* malaria in areas approaching elimination but stipulate that MTAT and focal test and treatment are not suitable interventions for reducing malaria transmission.^3,4^ Mass drug administration seeks to quickly reduce parasite biomass and prevent new infections for a certain period to rapidly reduce morbidity and mortality due to *P. falciparum* infection, requiring high coverage of vector-based interventions and good case management for success. A common MDA strategy uses dihydroartemisinin plus piperaquine (DHAp) to clear human infections and reduce the parasite reservoir, while providing longer chemoprophylactic protection against new infections.

Collectively, efforts have decreased malaria prevalence in Southern Province, Zambia, from 8% in 2012 to 0.6% in 2015,^5^ whereas overall deaths in Zambia attributed to malaria decreased 62% from 4,834 reported in 2010 to 1,827 in 2016.^6^ Much of this success is credited to a combination of sustained, high-level vector-based interventions, better surveillance, and improved access to malaria diagnosis and treatment through the scale-up of case management at health facilities and in villages through an increased number of community health workers. Clinical trials of population-level drug-based interventions were conducted in Southern Province, Zambia, including MTAT from 2011 to 2013, followed by MDA from 2014 to 2016. Outcomes from these trials have been reported.^7–9^ The MTAT study found that children in the intervention group had lower odds of a malaria infection than that of individuals in the control group (adjusted odds ratio = 0.47, 95% CI: 0.24–0.90) and that outpatient case incidence for malaria showed a modest decrease of 17% in the intervention group relative to that in the control group (incidence rate ratio = 0.83, 95% CI: 0.68–1.01).^8^ In addition to the high coverage of vector control and improved access to diagnosis and case management in the community, the MDA trial found that two rounds of MDA with DHAp rapidly reduced infection prevalence, infection incidence, and confirmed-case incidence rates, especially in low-transmission areas, with parasite prevalence decreasing from 7.7% at baseline to 0.5% after MDA in lower transmission areas, resulting in an 87% reduction compared with the control (adjusted odds ratio = 0.13, 95% CI: 0.02–0.92, *P* = 0.04).^7^ These declines were sustained after four rounds of MDA interventions, with a 72% (95% CI: 12–91%) reduction in malaria parasite prevalence as compared with those with the standard of care without any mass treatment.^9^ Thus, use of population-level, drug-based interventions, in the context of high coverage of vector-based interventions and improved treatment access, contributed to the decreased malaria burden observed between 2012 and 2016 in Southern Province, Zambia.

Rapid reduction in malaria prevalence and incidence through intervention use has been shown to dramatically change the parasite population structure.^10^ Declining transmission reduces parasite population genetic diversity as a consequence of reduced outcrossing and increased inbreeding.^11^ Genetic signatures that reflect reductions in effective parasite population size, population bottlenecks, and reduced transmission include a decrease in the complexity of infection (COI), the appearance of highly related and even genetically identical (clonal) parasites, and evidence of persisting parasite lineages across years.^10^ To assess the consequences of decreased malaria burden in Southern Province from 2012 to 2016 on parasite population structure, we compared the genotypes of parasites before and after application of drug-based interventions and performed a population genetic analysis. We expected that significant declines in malaria prevalence and incidence would correspond to a restricted parasite population that would exhibit decreased genetic diversity. Indeed, we observed strong evidence of a reduced parasite population with decreased COI including an increase in the proportion of infections that are monogenomic, highly related and clonal parasites after multiple rounds of MDA, and evidence for both focal and dispersed malaria transmission using genetic information from individual infections within and between households.

## MATERIALS AND METHODS

### Ethics statement.

Informed consent was obtained from all study participants. The study protocol was authorized by the Ministry of Health in Zambia and approved by the research ethics committees of the University of Zambia, PATH, and Tulane University (ClinicalTrials.gov: NCT02329301), and reviewed by the Human Research Protection Program of the Harvard T.H. Chan School of Public Health (IRB protocol 22688).

### Sampling scheme.

Malaria transmission in Southern Province is moderate, albeit heterogeneous, ranging from < 1% to > 50% in some areas,^7,12^ with an estimated overall prevalence of 14.9% and 20.3% among children younger than 5 years in malaria indicator surveys conducted in 2012^13^ and 2015,^14^ respectively. Transmission is highly seasonal, with the peak occurring from March to May. Rapid diagnostic test–positive samples were obtained from household-level data collection during two community-randomized trials conducted in the same geographical region of Southern Province between 2012 and 2016. Methods for the randomly selected household surveys and individual cohort are described in detail elsewhere.^7,8,15^ Dried blood spots on Whatman FTA filter paper (Whatman WB120205) were collected for all individuals in these two data collection activities. In brief, the first sample set (*n* = 836 children younger than 6 years) was collected during the peak malaria transmission season (April–May) in both 2012 and 2013 (baseline) as part of a community-randomized controlled trial designed to assess the impact of three rounds of an MTAT intervention that used RDTs for testing and artemether–lumefantrine for treatment.^8^ The surveys were standard 2-stage cluster designs with first-stage selection proportional to cluster sizes. The second sample set (*n* = 784 individuals ≥ 3 months) was obtained from an 18-month longitudinal cohort study, with monthly follow-up visits, conducted from December 2014 to May 2016 (cohort). The cohort was designed to evaluate a cluster-randomized controlled trial for assessing the impact of four rounds of community-wide MDA and household-level (focal) MDA (fMDA) with DHAp compared with that of no mass treatment (control).^7,9^ The analysis included comparisons of the differences between batches of survey (2012–2013) and cohort (2014–2016) samples.

For the cohort, two sample batches were created for genotyping and analysis, with the first batch representing samples collected between December 2014 and May 2015 and the second batch including samples collected between June 2015 and May 2016. Analysis was performed on these two batches or on sample sets based on the seasonality or intervention arm. Samples were grouped into sets corresponding to the first rainy season (season 1: December 2014–May 2015), dry season (intermittent; i.e., time between defined rainy seasons: June 2015–November 2015), and second rainy season (season 2: December 2015–May 2016). The cohort was recruited equally from the three different treatment arms: MDA, fMDA, and control.^7,9,12^ Clusters randomized to receive MDA and fMDA arms were administered the assigned intervention in four rounds (December 2014 [round 1], February 2015 [round 2], October 2015 [round 3], and February 2016 [round 4]).

### Genotyping.

Nucleic acid material was extracted from dried blood spots on filter papers collected from all RDT-positive individual observations (*n* = 1,620) detected during the two cross-sectional surveys and the longitudinal cohort. The genetic material was subjected to genotyping^16^ and the resulting data used for genetic analysis (Supplemental Figure 1 and Supplemental Table 1). Briefly, the dried blood spots were extracted for genomic DNA from 2 to 3, 6-mm punches using the manufacturer protocol from the Promega Maxwell DNA IQ Casework Sample kit (Promega AS1210, Promega Corp., Madison, WI).^17^ The samples were then subjected to pre-amplification^18^ and molecular barcode analysis.^16^ The genotypes were called by their base designation (A, T, G, or C) with missing alleles identified by “X” and working alleles where both the minor and major allele were designated by “N.”

### Data analysis.

A summary of decision-making for sample inclusion in the analysis is provided in the Supplemental Methods. In brief, we excluded from analysis those samples with missing data on more than four single nucleotide polymorphism (SNP) positions and identified these samples as having “failed” genotyping (see Supplemental Methods). We determined that samples with more than one site showing both fluorescent signals in genotyping (indicating that more than one allele was present) were “polygenomic” infections with more than one genome present in the patient sample. The remaining samples were referred to as “monogenomic” infections that contained only one parasite genome. We included all samples that “passed” genotyping for COI analysis and for calculating the proportion of infections that were monogenomic. Only monogenomic infections were included in the identity by descent (IBD) analysis and geographical comparisons. Household distances were calculated as the Euclidean distance between samples, expressed in kilometers (km), based on the recorded household latitude and longitude. Temporal distance was expressed as the difference in days between sample collection dates.

### Complexity of infection.

Complexity of infection analysis was carried out using THE REAL McCOIL method (Turning HEterozygous SNP data into Robust Estimates of ALelle frequency, via Markov chain Monte Carlo, and COI using Likelihood)^19^ accessed through the Broad.io/coil website. Significance was tested by comparing, as a test statistic, the square of the average difference in COI with an empirical distribution of the test statistic obtained by random subsampling (with replacement) pairs of samples, with a total of 551 and 354 samples representing the monogenomic infections from the surveys and cohort study, respectively, from the entire set of 905 pooled samples.

### Genotyping analysis.

Genotyping data representing the molecular barcode^16^ from all monogenomic infections were analyzed using a clustering tool (https://github.com/ndaniels/cluster-barcodes). Barcode groups are shown as nodes, with node size representing the number of samples with an identical genotype. Black and gray nodes represent cohort samples, and dark blue and light blue nodes represent baseline samples. Black and dark blue nodes indicate complete genotypes (i.e., with all 24 positions resolved), whereas nodes with highly related ambiguous barcodes (i.e., containing “X,” representing “no call” for missing data; or “N,” representing “multiple call” for more than one allele present in the sample) are represented with lighter shading. Edges are drawn between nodes whose barcodes have a Hamming distance of exactly one (“N” and “X” entries do not count toward Hamming distance).

### Identity by descent analysis.

Identity by descent was estimated using the software package hmmIBD.^20^ Default parameters were used, except that the maximum number of fit iterations was doubled to 10 because computation time was not an issue. The IBD estimate used was “fract_sites_IBD.” Pairwise analysis was carried out and the proportion IBD calculated (Supplemental Methods).

### Statistical analysis.

We estimated the failure/passage rates and monogenomic/polygenomic proportions based on the rules specified in the Supplemental Methods. Statistical analysis was performed to rule out any potential bias between sample sets due to genotyping failures that would impact conclusions about parasite population changes (Supplemental Methods). Baseline and cohort sample sets (and designated subdivisions of these sample sets) were assessed for significant differences using a Fisher exact test for small sample sizes, with a significance level of 0.05. A resampling analysis was performed to evaluate whether differences in average COI and distribution of COI were significant between baseline and cohort samples. Significance of the difference in average COI among the baseline and cohort sample sets was tested using the test statistic (*Mean*[*baseline samples*] – *Mean*[*cohort samples*])^2^ and comparing this with the empirical distribution of the test statistic obtained by repeatedly sampling with replacement pairs of samples of the same size as in the baseline and cohort sample sets. Sampling was from the pooled distribution of COIs in the baseline and cohort sample sets under the null hypothesis of no difference between baseline and cohort. Resampling was carried out in Mathematica 10.4.0 using the random choice algorithm.

## RESULTS

### Complexity of infection and proportion of monogenomic infections.

**Figure 1.**
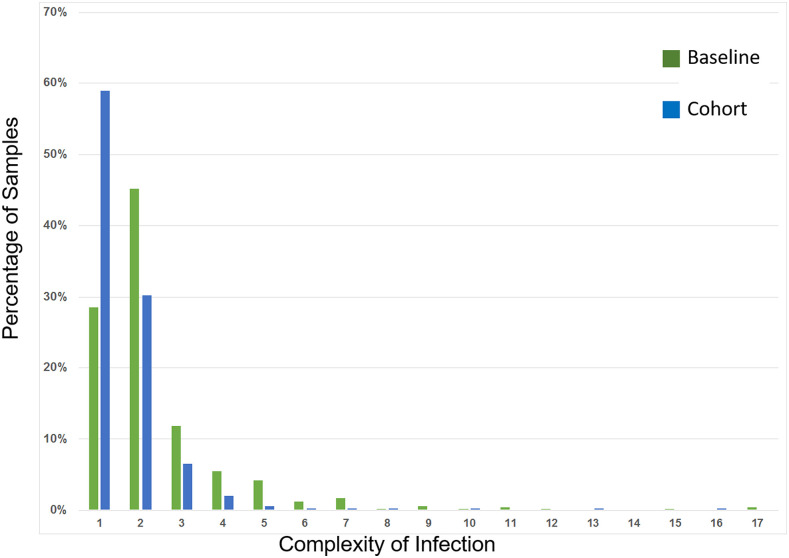
Distributions of complexity of infection for baseline and cohort samples. Genotype data from samples collected during the baseline (green) and cohort (blue) studies were analyzed for their complexity of infection (COI) using THE REAL McCOIL*.*^17^ The distribution of COI values for the two sample sets, with the percentage of samples (*y* axis) at a given COI value (*x* axis), is shown graphically. This figure appears in color at www.ajtmh.org.

A dramatic decrease in parasite genetic diversity, measured by COI and proportion of monogenomic infections, was detected between baseline (2012–2013) and cohort (2014–2016) sample sets. The average COI decreased significantly (*P* < 0.00001) from 2.38 (baseline) to 1.67 (cohort), coinciding with the scale-up of the scaled intervention package and the mass treatment interventions in the study area between 2014 and 2016 (Figure 1, Supplemental Table 2A). This decrease between baseline and cohort samples in the average COI was evident after accounting for potential sources of error, including increased genotyping failure rate among the cohort samples, and biases as a consequence of missing assays (Supplemental Methods). Comparison of infections from the baseline and cohort studies revealed a significant increase in the proportion of those infections that were monogenomic, from 14% (baseline) to 45% (cohort) (*P* < 0.00001) (Supplemental Table 2). The decrease in parasite population genetic diversity between the baseline and cohort samples implies a reduction in the potential for outcrossing (greater inbreeding) after application of population-level interventions.

Although a significant decrease in genetic diversity among parasites was observed between baseline and cohort samples, similar effects were not detected when the longitudinal cohort samples were interrogated based on seasonality (wet versus dry season), prevalence stratification (high versus low strata), or arm of intervention (MDA versus fMDA versus control), owing at least in part to the reduced statistical power of the comparisons due to a limited number of positive samples across time periods. For example, the observation that only 1.4% (5/354) of genotypes sampled from the longitudinal cohort were detected in the second rainy season (compared with 60.5% [214/354] from the first rainy season) precluded all comparisons of parasite population diversity between sample subsets (Supplemental Table 2A). Initial division of the cohort into batches (December 2014–May 2015 versus June 2015–May 2016) showed a significant increase in the proportion of monogenomic infections (*P* = 0.04, Supplemental Table 2B); but removal of 13 samples that lacked a collection date revealed no significant differences between these two sample sets (*P* = 0.06, Supplemental Table 2B). Comparison of samples from one malaria season to the next (December 2014–May 2015 versus December 2015–May 2016) showed no significant difference in the COI or proportion of monogenomic infections (*P* = 0.7), even though there was an approximately 48-fold decrease in the number of RDT-positive samples from the first (*n* = 484 RDT-positive samples) to the second (*n* = 10 RDT-positive samples) malaria season. Stratification of samples by high or low prevalence, with a break point of approximately 10% prevalence,^7,9,12^ also revealed no significant differences between sample subsets (Supplemental Table 2). Comparison of cohort samples based on the arm of intervention (e.g., comparison of batches 1 and 2 by arm: *P* = 0.2 [control]; *P* = 0.1 [fMDA]; *P* = 0.4 [MDA], Supplemental Table 2B) showed no significant differences in the proportion of monogenomic infections. Thus, changes in COI and proportion of monogenomic infections, consistent with reduction in parasite population diversity and parasite population constraint, were only detected when comparing baseline with cohort samples. These genetic trends mirror the epidemiological findings of a significant decline in parasite prevalence across a similar time frame and study population, where the overall parasite prevalence declined from 31.3% at baseline to 4.0% in 2016 at the end of the trial.^7^

### Evidence of highly related parasites.

**Figure 2.**
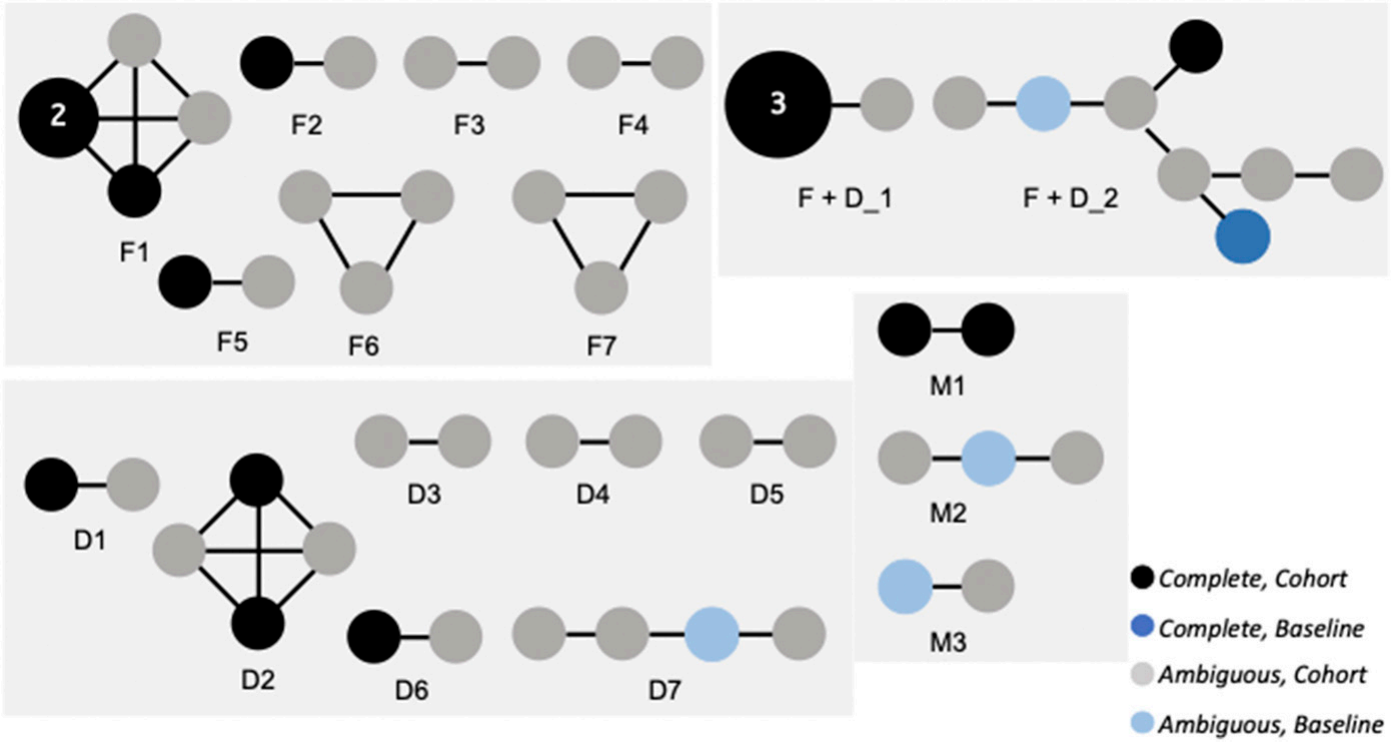
Genetic relatedness analysis of monogenomic infections from baseline and cohort studies. Groups of highly related parasites are shown. Saturated nodes (black or dark blue) represent samples with complete barcodes (all 24 assays), and transparent nodes (gray or light blue) represent samples with ambiguous barcodes (23 of 24 assays, containing either “X,” representing “no call” for missing data; or “N,” representing “multiple call” for more than one allele present in the sample). Black and gray nodes represent cohort samples, whereas outlined nodes (blank and lined) represent baseline samples. The size of the node indicates the number of samples, with all nodes having one sample, except for two nodes with either two or three parasites (indicated by a number and node size). Edges are drawn between nodes whose barcodes have a Hamming distance of exactly 1 (“N” and “X” entries do not count toward Hamming distance). Barcode group numbers (F1 – F7, F + D_1 – F + D_2, D1 – D7, and M1 – M3) match the barcode groups in Supplemental Table 3, with F indicating focal groups, D indicating dispersed groups, F + D representing a combined group with both focal and dispersed sample distributions, and M representing groups missing geographical data.

Highly related parasites, including genetically identical (clonal) samples, were evident in the cohort coinciding with the scale-up of the scaled intervention package and the mass treatment interventions in the study area between 2014 and 2016. Cluster analysis (Figure 2, Supplemental Table 3) and tests for IBD analysis (Supplemental Table 4)^20^ between infections were performed to test for the relatedness of parasite genotypes detected across the study. These analyses found no evidence of highly related parasites among baseline samples, consistent with these parasites representing a genetically diverse population based on the high COI value and relatively low proportion of monogenomic infections (14%) (Supplemental Table 2A). By contrast, analysis of the cohort samples revealed highly related infections as well as sets of clonal (barcode identical) parasites (see Supplemental Methods for definitions). Furthermore, we observed highly related parasites shared between the baseline and cohort samples. Cluster analysis of all monogenomic baseline and cohort samples (*n* = 248) identified 19 different highly related barcode groups with a Hamming distance of less than or equal to one (see Material and Methods) (Figure 2, Supplemental Table 3). These barcode groups were supported by IBD and identity-by-state analysis (Supplemental Table 4). For this analysis, highly related parasites were defined as having a Hamming distance of no more than one. Within these barcode groups were two sets of clonal parasites (Hamming distance of 0) identified among individuals after two rounds of mass treatment had been administered (detected between June and August 2015) (Supplemental Table 3). Four of the highly related barcode groups contained genotypes of parasites from the baseline study, with parasites sampled across a 3-year time span that crossed multiple transmission seasons (Supplemental Table 3). Detection of highly related parasites along with the identification of genetically identical parasites in Southern Province, Zambia, coinciding with the large decline in malaria transmission from 2012 to 2016 indicated that the overall parasite population was reduced and provided evidence of inbreeding, similar to patterns previously detected in malaria transmission settings that have decreased transmission to low prevalence levels.^10,17^

### Geographical analysis reveals both focal and dispersed transmission patterns.

**Figure 3.**
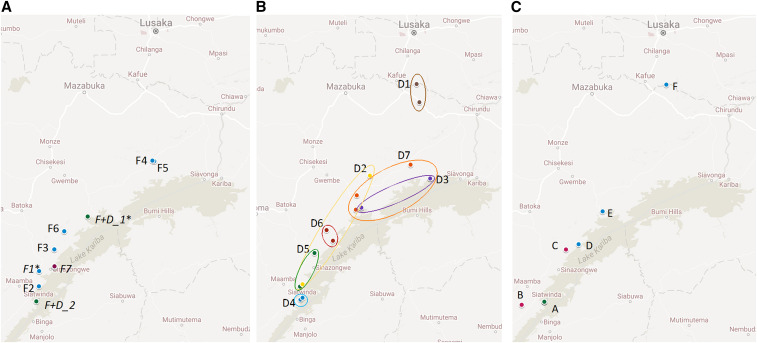
Geographical distribution of highly related infections reveals both focal and dispersed patterns. (**A**) Focal patterns of highly related infections include nine barcode groups of highly related parasites (indicated by F#), with either multiple highly related infections detected in a single household (all except F7) or barcode groups shared with or found in neighboring households (in italics: F7, F + D_1, and F + D_2). Two barcode groups contain clonal parasites (*: F1 and F + D_1). The three barcode groups shared with close neighboring households (italics: F + D_1, F7, and F + D_2) are at a distance that is either indistinguishable, 17 m apart, or within 1 km of one another, respectively. F + D barcode groups had both focal and dispersed patterns (see Figure 3C), and F + D_2 contained a highly related parasite identified in the baseline survey over 3 years earlier. (**B**) Dispersed patterns of highly related infections were observed for nine (including F + D, shown in Figure 3C) barcode groups with highly related infections within these parasite sets ranging from within 3 to 227 km (Supplemental Table 3). (**C**) An example of a highly related barcode with both focal and disperse patterns is seen for the F + D_2 group, which includes samples from both baseline (red: B and C) and cohort (green or blue: A and D–F) surveys. The maximal distance is 227 km (B to F), with one household (green: A) exhibiting multiple infections (“F + D_2” in Figure 3A, Supplemental Table 3).

Given the observation of highly related or genetically identical parasites in the cohort study, we analyzed the household and individual sources of the infections for possible patterns of transmission. Overall, two general patterns were detected, consistent with focal and dispersed transmission (Supplemental Table 3, Figure 3A). Focal transmission was supported by the detection of highly related infections within individual households (Figure 3A) or households within 1 km of each other. Half of the barcode groups with geographical information (8/16 = 50%) contained identical barcode or highly related infections within an individual household. Two focal barcode groups (F6 and F7) contained samples from a neighboring household, indicating tight geographic transmission foci (Figure 3A, Supplemental Table 3), with samples of barcode group F7 found among three different households in very close physical proximity (within 17 m) of each other. Further support of focal patterns of transmission include the identification of individuals repeatedly found with infections of the same highly related parasite group in half (4/8) of these households. This pattern may result from focal transmission of the same parasite genotype or failure to clear a parasite infection despite antimalarial drug treatment of the infected individual. Focal patterns of infection were mainly found on the same date within a household (6/8 = 75%) but did occur as far apart as 124 days (in one individual repeatedly positive for the same parasite barcode).

Evidence of dispersed transmission was provided by the detection of highly related parasite groups (9/16 = 56%) at geographic distances ranging from within 3 to 227 km apart (Figure 3B, Supplemental Table 3). Barcode-related infections that were dispersed and found in multiple households were identified within as few as 5 days of each other (D1) or as many as 314 days apart (F + D_2). Two of the eight barcode groups identified within households (F + D_1 and F + D_2) were also detected in another household at some distance (40 [F + D_1] and 227 [F + D_2] km, Figure 3C) from a household with multiple infections. For example, one individual (Person 1, Supplemental Table 3) was separately infected with two parasite genotypes. The first infection was of a focal barcode group shared with another household member (F3); the second infection that occurred 146 days later was part of a dispersed barcode group found in three households that were up to 40 km away.

## DISCUSSION

Genetic epidemiology detects parasite population changes in response to malaria interventions and reveals patterns of connectivity between infections that can inform intervention selection and targeting. The combination of a scaled standard of care intervention package and mass treatment interventions in the study coincided with large declines in malaria prevalence and incidence in Southern Province, Zambia,^7^ with an observed decrease in parasite population genetic diversity consistent with increased inbreeding. Parasite population constraint and inbreeding were evidenced by a significant decrease in COI and concordant increase in the proportion of monogenomic infections, along with the detection of highly related parasites, including barcode-identical samples, after multiple rounds of interventions. These changes in parasite population diversity occurred under the implementation of a comprehensive intervention package; that there was no impact of different intervention arms argues for the package as a whole rather than ascribing these declines to drug-based interventions alone. Mapping parasite infections among individuals and households revealed both focal and dispersed patterns of transmission with highly related infections focused within households as well as distributed across large geographic distances. Genetic signatures, thus, provide informative metrics for both tracking population-level changes and inferring the relationships between individual malaria infections.

Challenges to this analysis include an increase in genotyping failures among the cohort compared with baseline samples and a lack of infection-positive samples in the second season of the longitudinal cohort collection due to the dramatic reductions in malaria in the study area.^9^ Technical or biological reasons may explain the increased number of cohort samples that could not be successfully genotyped. There were no differences in the extraction and genotyping methods for the two sample sets, and quality control metrics for extraction efficiency and assay performance were met throughout sample processing. In addition, false-positive findings may result from repeatedly clearing parasite infections while retaining antigens that could produce a positive diagnostic test finding in the absence of an infection. This has been noted previously, in which the false positivity rate for RDTs increased in the second half of a study carried out in a low transmission region.^21^ To address the potential impact of these failures on the conclusions drawn from our analysis, we assumed a worst-case scenario in which all excess failures over baseline had a COI distribution the same as that in baseline, and adjusted the average COI in the cohort set under this assumption. The difference in the COI between the baseline and cohort sample sets was significant with or without adjusting the mean under these worst-case assumptions. Thus, despite increased failures among cohort samples, there was a significant decrease in the COI from before (baseline) to after (cohort) the MDA intervention in Southern Province.

The inability to detect differences between sample sets from the longitudinal cohort based on seasonality, level of transmission, or arm of intervention may be accounted for by dramatic reductions (up to 50-fold) in the number of RDT-positive infections available for analysis from one malaria season to the next across all intervention arms of the longitudinal cohort.^9^ Indeed, among the 354 successfully genotyped samples in the longitudinal cohort, only five (1.4%) of these were detected in the second malaria transmission season (compared with 214 or 60.4% in the first malaria season, 122 or 34.5% detected in the intermittent season, and 13 or 3.7% assigned no season [Supplemental Table 2A]). Heterogeneity in transmission levels across the cohort may also have impacted the analysis because the majority of infections detected (303/354 = 86%) were from the high prevalence regions and no positive RDT samples were genotyped from the low transmission regions during the second malaria season. Moreover, previous reports suggest the lack of sensitivity of RDTs in areas with low transmission due to infections with low parasite density.^21,22^ Nevertheless, dramatic differences in parasite prevalence across the study period (2012–2016) significantly impacted the parasite population structure by decreasing genetic diversity across this time in Southern Province.

Reduction of parasite population diversity (i.e., decreased COI and increased monogenomic infections) was accompanied by the appearance of highly related and even clonal parasites after the scaled intervention package and mass treatment campaigns were applied in the study area. These highly related parasites indicate a reduction in the potential for outcrossing or greater inbreeding among the cohort study samples. Although we did not detect these highly related parasites in the baseline samples alone, when we analyzed all samples together, we detected four genotypes from among the baseline samples that share barcode genotypes with the cohort infections, suggesting that some of these highly related parasite lineages have been retained for more than 3 years. The survival of specific lineages may indicate these parasite types have a fitness advantage, such as is observed in Southeast Asia, where drug-resistant parasites have emerged and dominated the surviving parasite population.^22,23^ We have previously observed a similar pattern in Senegal, where reduced transmission has been accompanied by both an increase in clonal parasite populations and the persistence of clonal lineages.^10,17^ Detection of genetically related infections in Zambia is consistent with a decrease in malaria transmission coincident with the application of the scaled intervention package plus mass treatment, which suggests that patterns originally detected in Senegal may be broadly applicable and that genetic signatures are relevant across a range of transmission levels. These genetic signatures were found in Senegal with prevalence values as low as 1%^24^ and in Southern Province, Zambia, with prevalence values as high as 15%.^13^

Genetic “fingerprinting” of specific parasite infections, coupled with information about the households, the individual, and the date of infection, allows for the mapping of relationships between infections during the scale-up of aggressive intervention in the Zambia study area. Genetic data provide evidence for both focal and dispersed patterns of infection in this area. Focal infections accounted for about half of the highly related and clonal parasite groups, which were localized to individual or very geographically proximal households. Dispersed infection patterns accounted for the remaining half of the highly related barcode groups and were distributed across as much as 227 km and as long as 314 days between infections (excluding baseline samples). Several of these dispersed infections occurred along Lake Kariba and might have resulted from movement of infected individuals over longer distances via fishing boats. Interesting patterns included repeated sampling of highly related and barcode-identical parasites in specific individuals, either because of failure to clear the original infection or as a consequence of repeatedly being infected with the same parasite barcode group. Finally, some barcode groups had hybrid patterns, with both focal (within a household) and dispersed (shared across both time and space). Coupled with data about location and time, genetic information can be used to describe the relatedness between infections and can begin to map transmission linkages. Genetic data can also be used to identify the origin or source of infections, address questions of whether an infection is local or imported, or detect whether specific dominant parasite types (i.e., as a consequence of drug resistance) are evident in a population. With this information, the basic reproductive number across geographic areas over time can be estimated.^10^ Thus, genetic information provides more granular information that reveals potential patterns of transmission that can inform how interventions are working, what interventions should be used, and where best to target these interventions for greatest impact. For example, if there is evidence that most infections in a very low transmission area are due to importation, then this might support the use of case investigation. Alternatively, if there is evidence for local transmission, then the use of vector-based interventions would be warranted.

Efforts to reduce malaria burden across the transmission spectrum require ways to measure the impact of interventions on transmission. Traditionally, changes in prevalence or incidence have been used to assess intervention impact,^11^ but as transmission becomes very low, precise estimates of prevalence and incidence become more difficult. Genetic methods not only offer an opportunity to validate traditional metrics but also provide more fine-grained information about the relationship between infections, thereby helping to describe patterns of transmission when levels are low. Previous studies have detected dramatic changes in parasite population genetic signatures over time in Senegal following the implementation of major interventions.^10,17^ In this context, evidence for both transmission decline and rebound was identified using epidemiological modeling.^10^ The use of genetic measures can both detect population-level changes and track individual parasite patterns in low or very low malaria transmission settings. At the population level, genetics can detect changes due to population bottlenecks and identify persistence of parasite lineages. At the individual level, genetic “fingerprinting” can track individual parasite types with the potential for genetic relatedness to help map transmission networks. Thus, the use of genetic signatures, along with clinical and epidemiological data, can help guide decision-making related to the reduction of malaria burden.

## Supplemental figure, tables and methods

Supplemental materials
