# Supplementary figures and images for: Evidence for Reduced Malaria Parasite Population after Application of Population-Level Antimalarial Drug Strategies in Southern Province, Zambia
Source: Am J Trop Med Hyg. 2020 Jul 2;103(2 Suppl):66–73. doi: 10.4269/ajtmh.19-0666 (PMC7416975; doi:10.4269/ajtmh.19-0666)

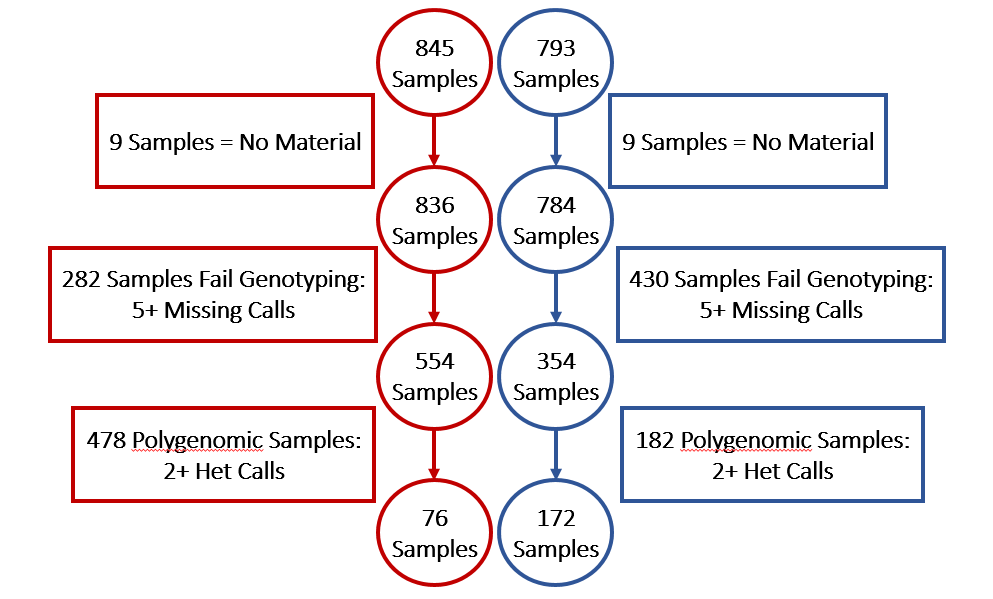

Supplement: Supplementary file 1 [file tpmd190666.SD1.tif]
