# Supplementary material for: Evidence for Reduced Malaria Parasite Population after Application of Population-Level Antimalarial Drug Strategies in Southern Province, Zambia
Source: Am J Trop Med Hyg. 2020 Jul 2;103(2 Suppl):66–73. doi: 10.4269/ajtmh.19-0666 (PMC7416975; doi:10.4269/ajtmh.19-0666)
Supplement: Supplementary file 2 [file tpmd190666.SD2.docx]

***Supplemental Methods***

*Rules:*

*Allele Calling:* Genotypes are called by their base designation for DNA: A, T, G, or C. Missing alleles are indicated by an “X”; and, working alleles that are called biallelic (i.e., both the minor and major allele are called by genotyping) are designated by an “N”.

*Pass/Fail:* Any 24 single nucleotide polymorphism (SNP) barcode that is missing 4 or fewer assays is included in the analysis and considered as having “passed” genotyping. This means that barcodes missing 0, 1, 2, 3, or 4 assays are included in the “passed” samples. Any sample missing 5 or more assays of the 24 SNPs in the barcode are called “failed” samples.

*Monogenomic/Polygenomic:* Any 24 SNP barcode genotype that has “passed” and has one or fewer “N” calls is classified as “monogenomic”. This means that any passed sample with 0 or 1 N assays of the 24 SNP barcode is called monogenomic and any passed sample with 2 or more N assays of the 24 SNP barcode is called polygenomic.

*Identical Genotypes:* an identical genotype is one where all 24 SNPs are called and they match perfectly (i.e., at all 24 SNP positions of the barcode) between 2 samples.

*Highly Related Genotypes:* a highly related genotype is one where 23/24 SNPs are called and they match perfectly at all of these 23 positions between the 2 samples. This allows for one of these positions to “not match” or be different; one of these positions to have missing data; or, one of these positions to be called “N”.

*Bias Evaluation for Missing Data:*

While more than half (908/1620 = 56%) the samples yielded a genotype (Supplementary Table 1), there were significant differences in genotyping success observed between sample sets (Supplementary Table 2). Greater genotyping success was obtained for cross-sectional baseline samples, compared to longitudinal cohort samples. Statistical analysis using a resampling strategy was used to rule out any potential bias due to sample failures that would impact conclusions about parasite population changes as a consequence of population-level drug use for these samples (Material and Methods). Genetic analysis was therefore performed on samples collected before and after population-level drug interventions with the caveat that the conclusions are derived only from the available data.

*COI analysis:* A potential source of error comes from the fact that the cohort samples had a failure rate of 55% versus 34% among the baseline samples (Supplemental Table 1). The excess failure rate of 21% among 784 cohort samples corresponds to 165 excess missing samples in the cohort data. In the worst-case scenario, all of these would have a distribution of COI equal to that in the baseline set, resulting in a downward bias in the mean of the samples recovered in the cohort study. Correcting for this worst-case bias would increase the mean COI of the cohort samples by about 14%, from 1.67 to 1.89. However, resampling tests of 2.38 (baseline) versus 1.89 (worst-case cohort) indicates a *P* value of about *P* ≈ 10^−4^. This worst-case analysis reinforces the conclusion that the cohort samples have a significantly lower COI than the baseline samples, however we emphasize that we have no reason to suspect that the failed samples in the cohort study do in fact have an average COI like that in the baseline study.

*Sample Filtering Analysis:*

Here we describe the decision-making process for our analysis. We first filtered the dataset to include only samples with ≤ 10 failed assays and ≤ 1 heterozygous call, the latter to eliminate samples with clear evidence for multiple genomes [i.e., complexity of infection (COI) > 1]. Sample quality evidently varied between the two data subsets (Baseline and Cohort), based on the distribution of missing assays (Supplemental Methods, Table 1).

*Supplemental Methods Table 1. Missing assays, for all samples with ≤* *1 heterozygous call.*

We then examined this dataset to see whether samples that had a heterozygous call included a substantial number with COI > 1, but did not find good evidence for this possibility. If heterozygous calls represent genuine heterozygous positions, their occurrence should be strongly correlated with the allele frequency in the population for that assay. Instead, little correlation was evident (Supplemental Methods, Figure 1). What we did find was a marked enrichment of missing data in samples with a heterozygous position (1.3-fold in the cohort set, 1.5-fold in the baseline set), suggesting that such samples were generally of poorer quality, and that the heterozygous calls largely represented experimental artefacts. As a result, we included samples with a single heterozygous call in subsequent analysis.

To assess whether there was a bias introduced because of the increased failures, we selected only samples with 0 or 1 assay missing.

*Supplemental Methods, Figure 1. The heterozygosity call rate for each assay, plotted as a function of that assays minor allele frequency in the entire dataset.*

We next determined the minimum number of successful assays to require for a sample, in order to effectively discriminate between non-identical samples. For this purpose, we calculated the fraction of pairwise comparisons that were identical, first for samples with no missing data, and then for samples with increasingly large numbers of failed/heterozygous assays; what we were interested in was the marginal rate of pairwise identity, i.e. identity among previously untested pairs as new samples were added. The rate of pairwise identity is independent of sample size, so an increase in its value as less informative samples are added implies a loss of discriminating power between samples. We found that the rate began to increase significantly when the number of good assays fell below 19 (Supplemental Methods, Figure 2). (This was also the point at which we began to observe samples that belonged to multiple, inconsistent clusters of identical samples.) We accordingly used this as the minimum number of valid genotypes.

*Supplemental Methods, Figure 2. Effect of missing data. Plotted is the rate of identical sample pairs (as a fraction of all sample pairs)*

Finally, we addressed a pair of related questions about the meaning of highly similar samples: (1) whether high similarity between samples actually represents high relatedness; (2), assuming it does, whether degrees of relatedness among highly similar samples can be distinguished. To investigate the first question, we carried out a simulation of our barcode data, with the goal of determining whether there are more highly related sample pairs than we would expect by chance. We took the full dataset (filtered as above), retained the existing pattern of missing/heterozygous genotype calls, and replaced the real genotypes with randomly chosen alleles, distributed according to the population allele frequencies for the assay in question. We analyzed 100 simulated datasets that were generated this way.

In order to extract the maximum information about relatedness from the barcode data, for this analysis we processed both simulated and real data with hmmIBD, a software package for inferring identity by descent in haploid genomes, as this permits us to incorporate knowledge of the allele frequencies of the assays into the estimate of overall identity between samples.

The simulation gives good overall agreement with the data (Supplemental Methods, Figure 3). In real data there was a clear excess of sample pairs with very high relatedness (Supplemental Methods, Figure 4), however. We take the discrepancy to be evidence that the bulk of highly related samples are in fact closely related.

For insight into the second question, we compared sample pairs that were identical to those that differed by a single genotype call. Nearly identical pairs showed a strong signal of having poorer sample quality than identical pairs (Supplemental Methods, Figure 5), based on the number of missing assays, suggesting that many of the former were likely also identical. We therefore opted to treat “highly related” as a single category, rather than attempting to distinguish between more and less related pairs.

*Supplemental Methods, Figure 3. Comparison of simulated and real barcode data for the Zambia dataset. Shown is the distribution of inferred relatedness (described by the estimated fraction of the genome that is identical by descent) for all pairs of samples.*

*Supplemental Methods, Figure 4. Comparison of simulated and real data, zoomed. Cohort data only are shown.*

*Supplemental Methods, Figure 5. Barcode quality for identical pairs, compared to quality for nearly identical pairs.*
